# Case Report: Bronchial splinting for the treatment of compressive tracheobronchomalacia

**DOI:** 10.3389/fcvm.2026.1904274

**Published:** 2026-09-22

**Authors:** Xingchen Lian, Binghe Wang, Quanwei Zhu, Yu Chen, Peipei Chang, Ping Wen, Lin Ma, Yuhang Liu

**Affiliations:** 1 Department of Cardiovascular Surgery, Dalian Women and Children’s Medical Group, Dalian, China; 2 Graduate School, Dalian Medical University, Dalian, China; 3 Heart Center, Beijing Children’s Hospital, Heilongjiang Hospital, Harbin, China

**Keywords:** bronchial splinting, case report, congenital heart disease, pediatric, tracheomalacia

## Abstract

Compressive tracheomalacia is a rare subtype of pediatric airway malacia. We report a case of compressive tracheomalacia treated with bronchial splint implantation. No significant complications occurred during the 2-year follow-up, and the child maintained normal growth and development. Bronchial splint implantation is a safe and effective therapeutic option for such cases and can markedly improve respiratory function in children with favorable long-term tolerance.

## 1 Introduction

Tracheobronchomalacia is a common cause of pediatric airway obstruction, characterized by weakened structural support of the tracheal wall due to congenital developmental abnormalities or external compression (1). Current treatments for compressive tracheobronchomalacia include tracheoplasty, aortopexy, endoluminal tracheal stenting, and bronchial splinting (2). Each surgical approach has its respective advantages and disadvantages. This report describes a 6-month-old infant with severe compressive tracheobronchomalacia induced by cardiomegaly secondary to a large atrial septal defect. The patient underwent concurrent cardiopulmonary bypass-assisted cardiac defect repair and biodegradable poly-L-lactic acid (PLLA) external tracheal stent implantation to relieve airway compression. The patient achieved substantial anatomical and functional recovery of the airway structure. This study evaluates the clinical value and long-term safety of external tracheal stenting in the management of compressive tracheobronchomalacia of a cardiac origin in pediatric populations.

## 2 Case description

A 6-month-and-4-day-old female infant was admitted with a 1-month history of recurrent cough. The patient presented with a persistent non-febrile cough without wheezing or dyspnea. She was previously diagnosed with bronchitis at a local hospital and received anti-infective therapy and nebulized bronchodilator treatment, which achieved only temporary symptom relief with frequent recurrence. On admission, vital signs were as follows: temperature, 36.7 ℃; heart rate, 135 beats/min; respiratory rate, 33 breaths/min; and body weight, 7.8 kg. Physical examination revealed a precordial systolic murmur and diminished breath sounds in the left lung, with no bilateral pulmonary rales. The patient's family declined genetic testing. No remarkable past medical or family history was reported.

Admission chest radiography demonstrated left pulmonary atelectasis. Cardiac ultrasonography revealed a secundum atrial septal defect, moderate mitral and tricuspid regurgitation, pulmonary hypertension, and unclear visualization of the left pulmonary veins, with preserved left ventricular function (Figure 1). Contrast-enhanced chest computed tomography (CT) further confirmed right atrial and ventricular enlargement with a 7.7-mm atrial septal defect (Figure 2). Additional imaging findings included left pulmonary atelectasis, right pulmonary exudation with focal emphysema, and severe compressive occlusion of the left main bronchus (Figure 3). Fiberoptic bronchoscopy was performed to identify the etiology of left lung atelectasis. Fiberoptic bronchoscopy demonstrated severe stenosis and limited patency of the left main bronchus (Figure 4). The left main bronchus was externally compressed with a straight linear configuration. No substantial changes in the luminal caliber were observed between the inspiratory and expiratory phases during spontaneous respiration. A 2.8-mm outer-diameter bronchoscope could only pass through only with applied external force.

**Figure 1:**
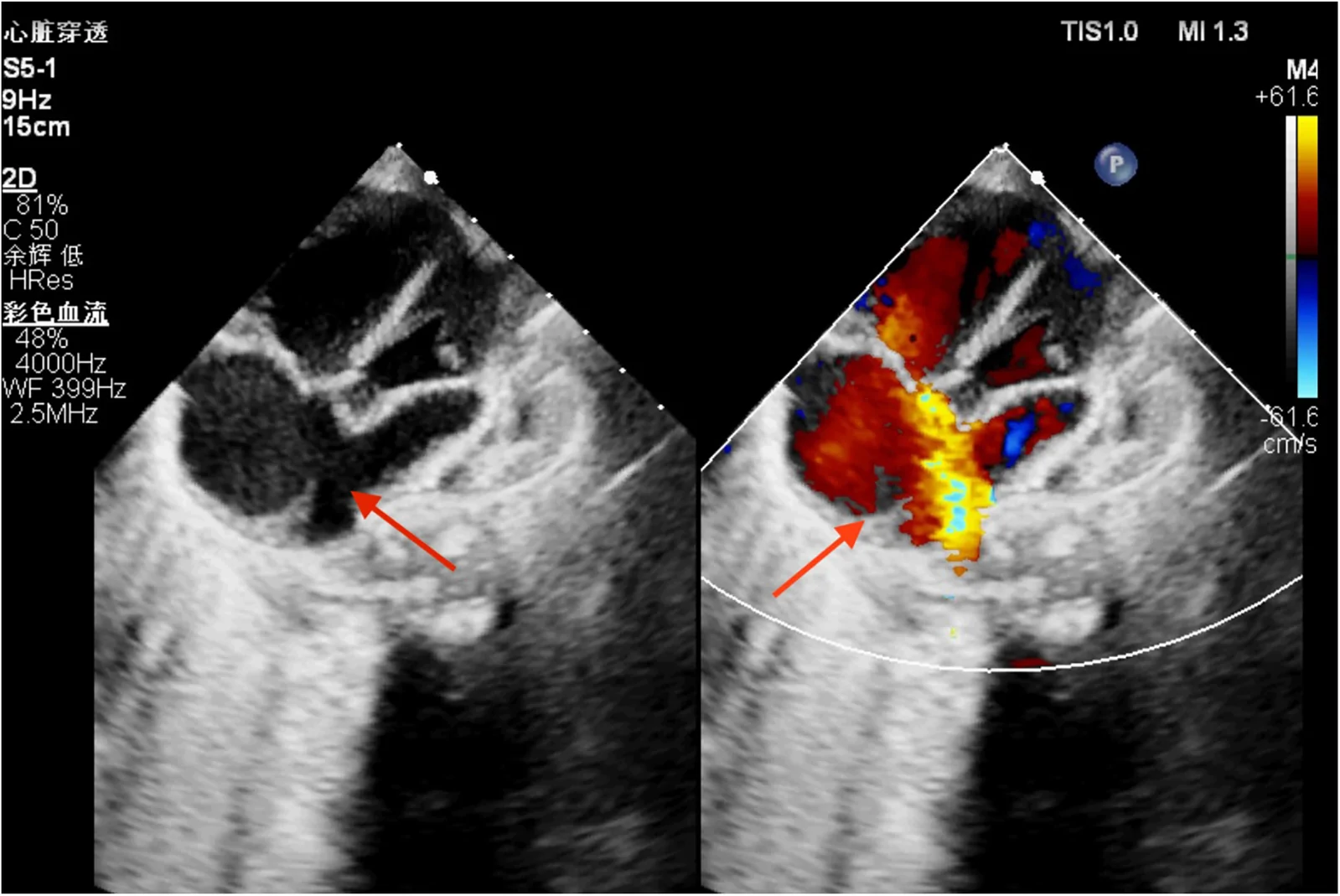
Cardiac ultrasonography revealing an atrial septal defect, as indicated by the arrow.

**Figure 2:**
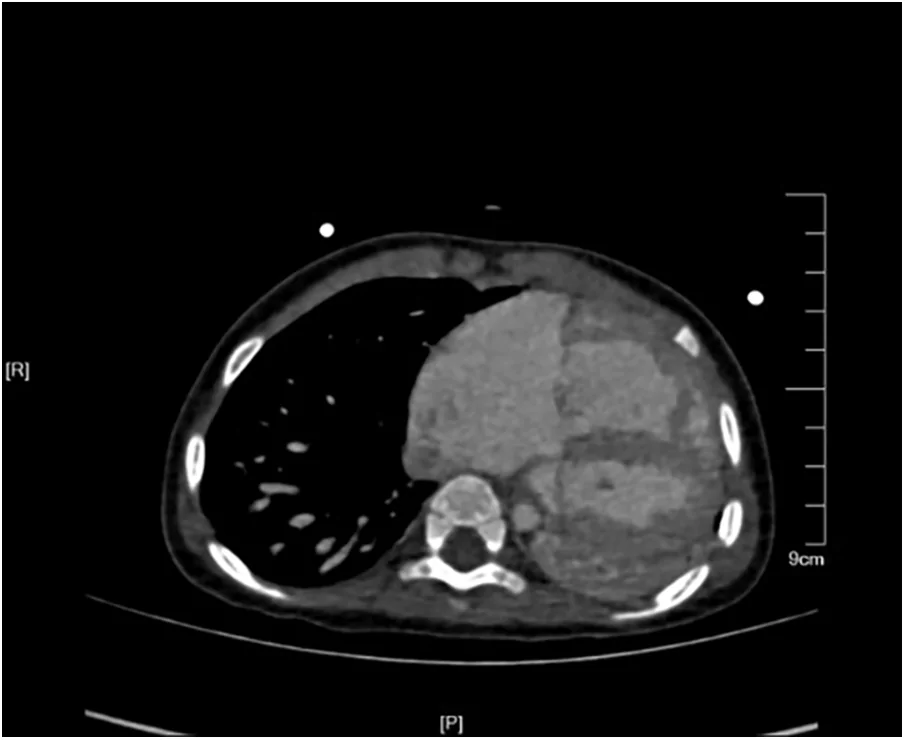
Cardiac-enhanced CT showing significant cardiac enlargement.

**Figure 3:**
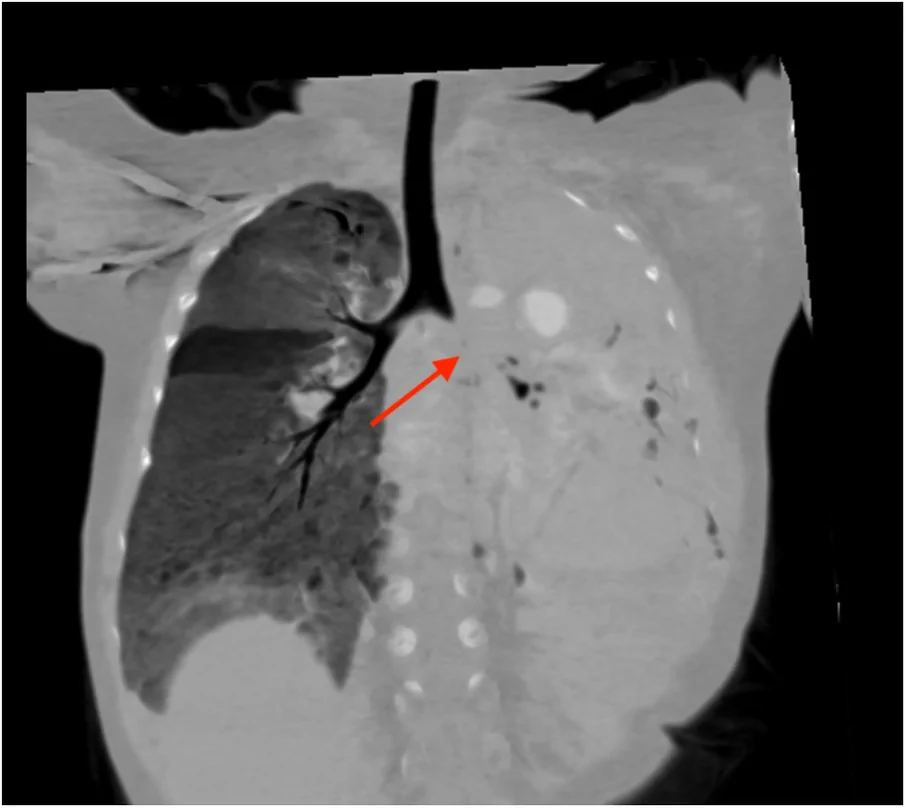
Chest CT coronal imaging showing significant compression of the left main bronchus, as indicated by the arrow.

**Figure 4:**
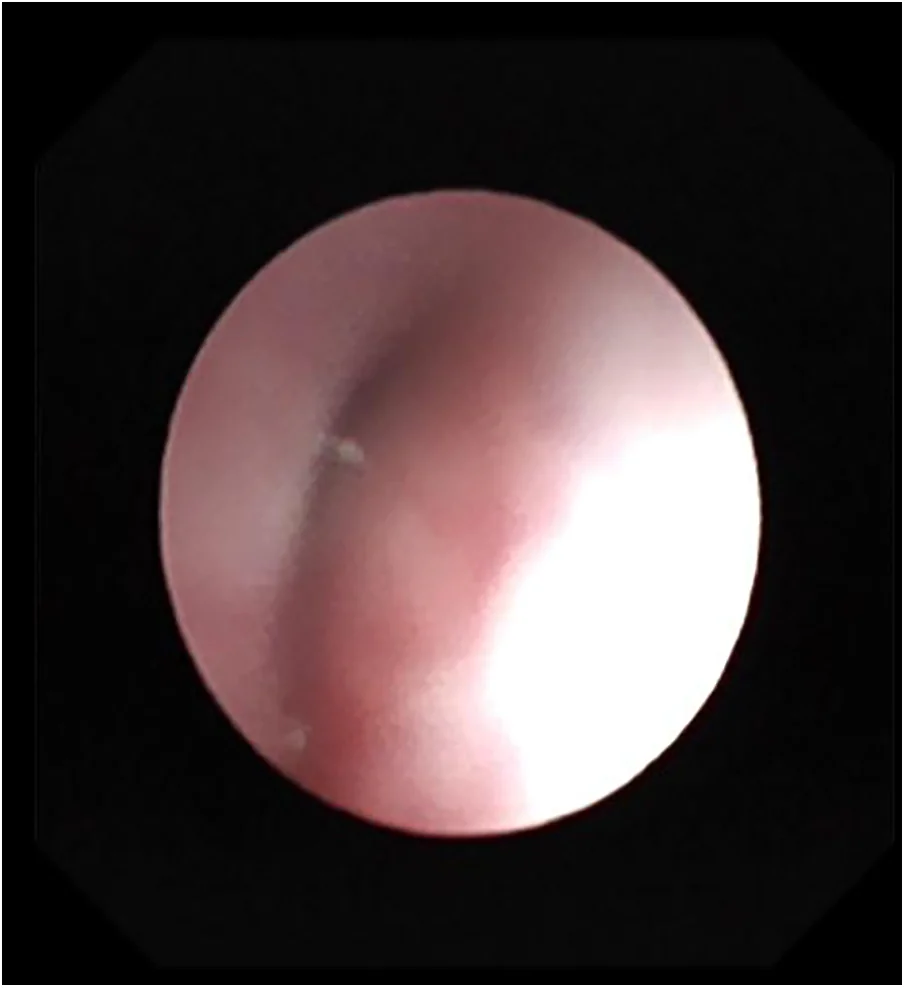
Fiberoptic bronchoscopy demonstrating severe stenosis and limited patency of the left main bronchus.

After multidisciplinary consultation and comprehensive evaluation, tracheomalacia was confirmed to be caused by compression from the enlarged cardiac structures and blood vessels. Surgical relief of the tracheal compression combined with intracardiac malformation repair was indicated, with no absolute contraindications to bronchial splint implantation.

The definitive surgical plan consisted of cardiopulmonary bypass-assisted atrial septal defect repair combined with bronchial splint implantation under intraoperative bronchoscopic guidance to facilitate accurate stent placement.

## 3 Surgical procedure

The patient underwent median sternotomy, cardiopulmonary bypass-assisted atrial septal defect repair, and simultaneous external tracheal stent implantation. The trachea and surrounding soft tissues were extensively dissected to expose the malacic segment, and the exact lesion location and length were confirmed via real-time fiberoptic bronchoscopic guidance.

A biodegradable PLLA microplate was customized for external stenting. Under standardized heating conditions (preheating at 95–105 ℃ for 2–3 min, followed by 6–8 s of formal heating), a metal measurement model matching the anatomical lesion was fabricated intraoperatively. A U-shaped PLLA microplate was molded according to individual bronchial parameters. The outer diameter of the stent was designed to be 3 mm larger than the normal bronchial diameter, and the plate length and width were determined based on the bronchial circumference and the longitudinal length of the malacic segment.

After edge trimming, the PLLA plate was softened in a warm water bath at 70 ℃. Once pliable, the plate was molded around a graded Hegar dilator with a reserved gap to facilitate fitting onto the bronchial surface. After cooling and stereotyping (Figure 5), the U-shaped stent maintained stable structural integrity and was easily detached from the dilator for implantation.

**Figure 5:**
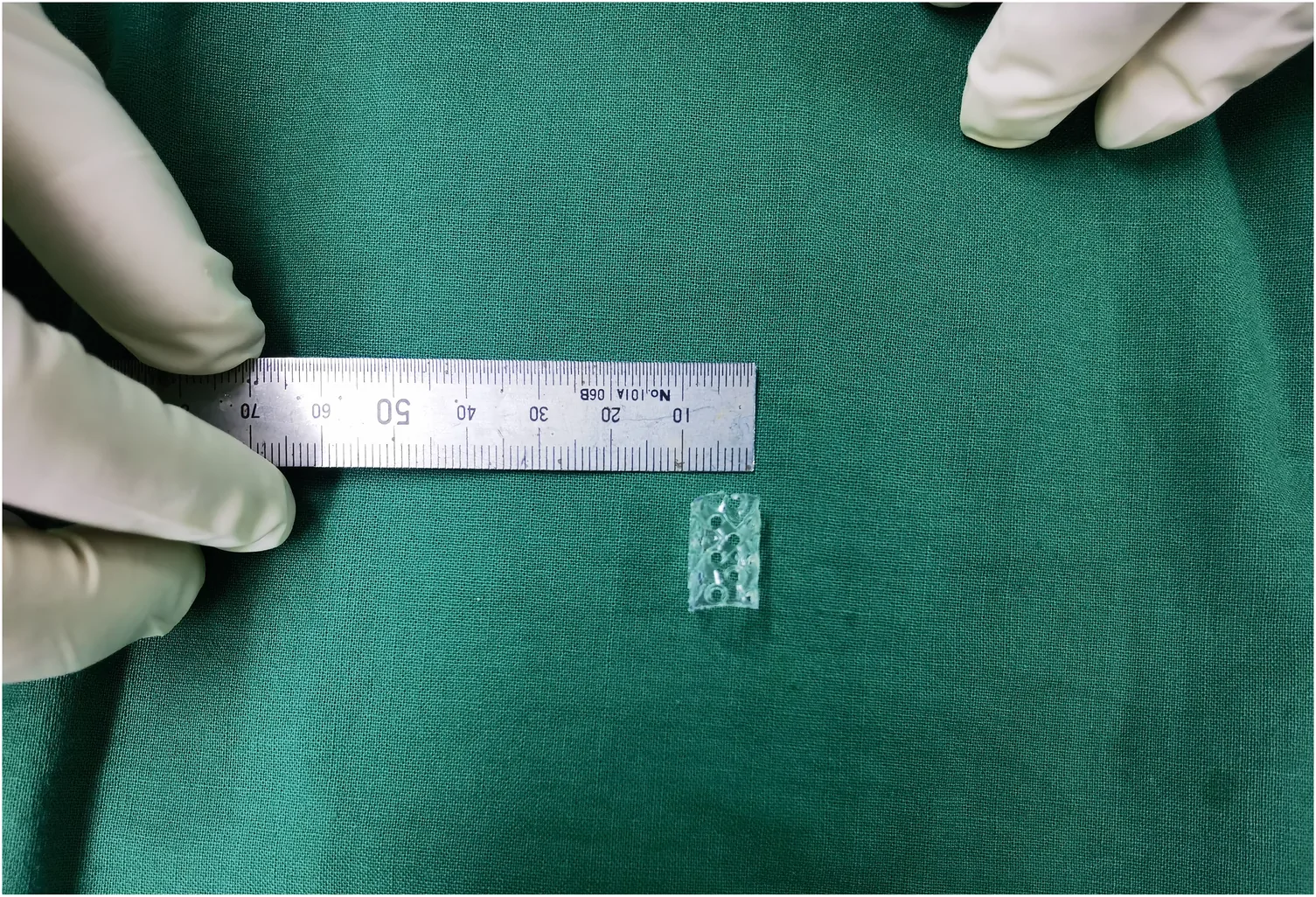
Tracheal external stent shaping.

The customized U-shaped stent was placed externally over the cartilaginous layer of the malacic bronchus (Figure 6). Preset sutures were threaded through plate holes and tied to fix the bronchial adventitia to the microplate, effectively expanding the stenotic airway lumen. Intraoperative bronchoscopy immediately confirmed complete resolution of cardiac-induced airway compression (Figure 7). After stent placement, autologous pericardium was loosely wrapped around the PLLA microplate to reduce friction on adjacent tissues and allow physiological bronchial cartilage growth. Standard atrial septal defect repair was subsequently completed uneventfully.

**Figure 6:**
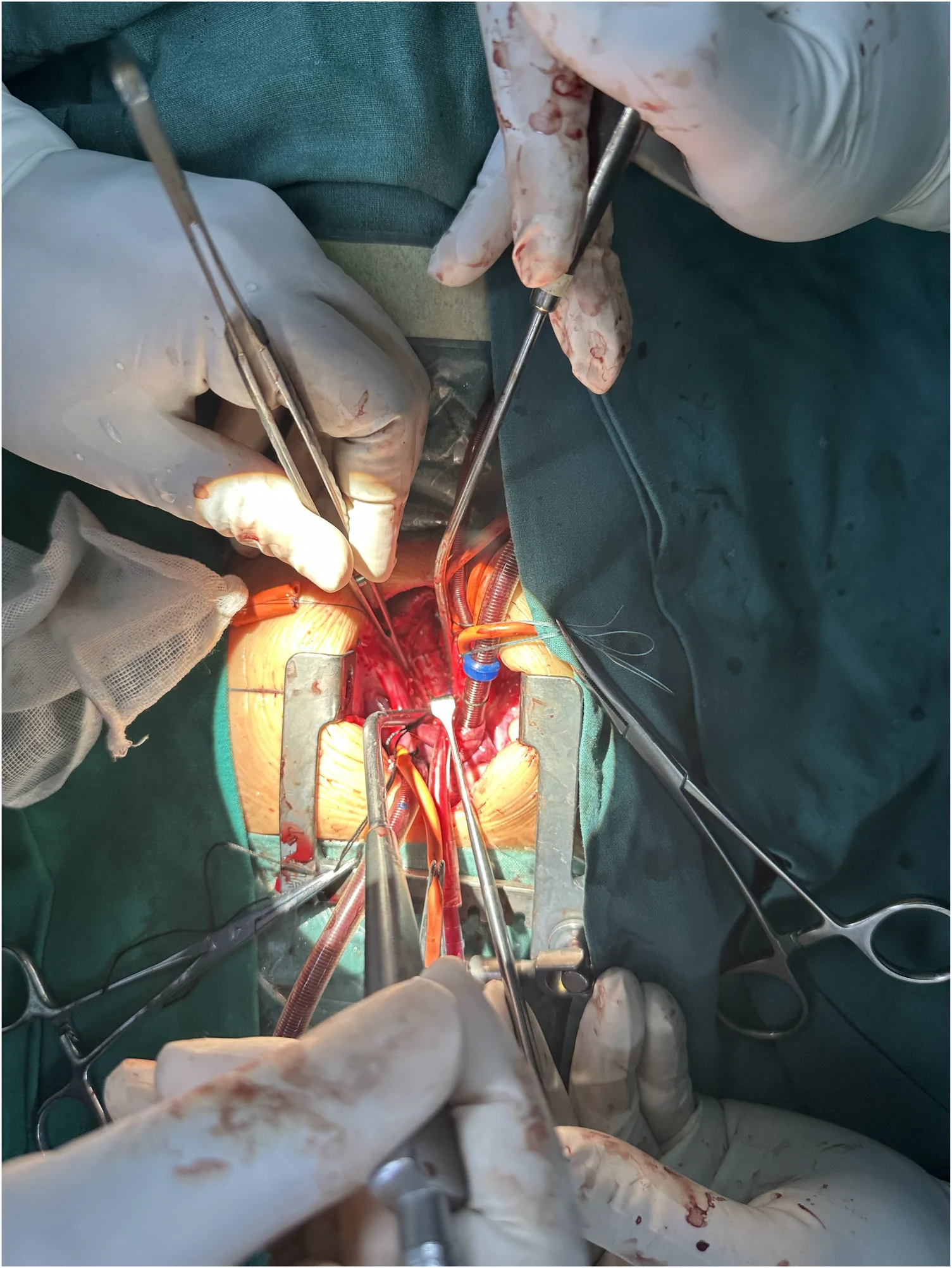
Implantation of an external tracheal stent during the operation.

**Figure 7:**
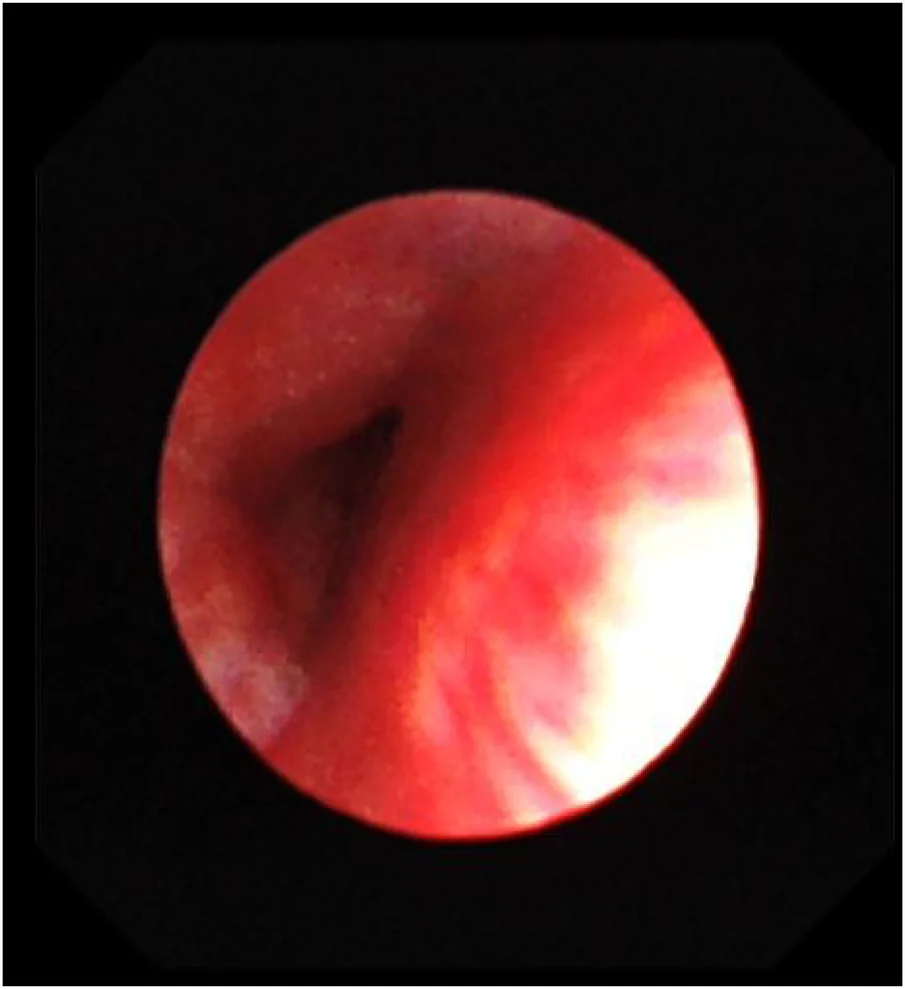
Postoperative fiberoptic bronchoscopy examination indicating that the compression of the left main bronchus has significantly improved.

The patient was transferred to the pediatric intensive care unit after surgery and received standardized postoperative management, including mechanical ventilation, antibiotic prophylaxis with ceftriaxone, hemostatic treatment, and nutritional support. Chest radiography at 24 h postoperatively showed substantial resolution of the left pulmonary atelectasis. Endotracheal intubation was removed 48 h after surgery, and the patient maintained stable respiration with nasal catheter oxygen inhalation. The patient recovered uneventfully and was discharged on postoperative day 7.

Structured follow-up was performed at 1, 3, and 6 months postoperatively. Serial chest CT examinations confirmed stable splint position without displacement or deformation throughout the follow-up period. The tracheobronchial lumen presented normal morphology without expiratory collapse, and the airway mucosa remained smooth without inflammatory hyperplasia or granulation tissue formation. The patient remained asymptomatic, without recurrent cough, dyspnea, or wheezing, and achieved normal age-matched growth and development at the 2-year follow-up endpoint.

## 4 Discussion

Tracheobronchomalacia is a common cause of pediatric airway obstruction. It is defined as reduced tensile strength of the tracheal wall resulting from congenital dysplasia or acquired factors, leading to expiratory airway collapse and clinical manifestations such as dyspnea, wheezing, and chronic cough. Congenital cases are mostly associated with tracheal cartilage hypoplasia. Acquired etiologies commonly include peritracheal tumors, infection, trauma, vascular anomalies, and cardiomegaly. Long-term external compression induces degenerative changes in the tracheal cartilage and impairs its supportive function (3).

Bronchoscopy is regarded as the standard for diagnosing tracheobronchomalacia, enabling direct visualization of tracheal lumen morphology, collapse severity, and mucosal conditions, alongside accurate localization and assessment of the lesion extent (4). Contrast-enhanced chest CT combined with three-dimensional tracheal reconstruction clearly delineates the anatomical relationship between the trachea and adjacent structures (such as the heart and great vessels), clarifies the cause of compression, and provides critical evidence for diagnosis and treatment planning. The combined application of these two examinations improves diagnostic accuracy and reduces the number of missed and misdiagnosed cases.

Treatment strategies for pediatric tracheobronchomalacia are individualized according to etiology, stenosis severity, and patient age. The disease is graded based on luminal reduction: mild (<1/3 reduction), moderate (approximately 1/2 reduction), and severe (≥4/5 reduction or near-complete occlusion) (5). Mild cases are managed conservatively with infection prevention and nebulized bronchodilators and glucocorticoids. For moderate-to-severe stenosis refractory to conservative treatment, surgical intervention, including tracheoplasty, endoluminal/external stenting, and etiological decompression surgery, is mandatory.

Endoluminal stenting is suitable for treating severe focal tracheal stenosis. However, the delicate pediatric airway mucosa is vulnerable to chronic stent-related irritation, which may lead to mucosal injury, secondary infection, and stent displacement. Moreover, stent replacement surgery may be necessary as children grow due to size mismatch (6). Although aortopexy is widely performed, it requires extensive tissue dissection and cannot completely resolve compression caused by other cardiac structural abnormalities. Furthermore, repeat surgery is technically challenging, and prolonged postoperative recovery may lead to persistent airway stenosis. Bronchoplasty cannot address compressive tracheal stenosis (7–11).

External tracheal stenting provides mechanical airway support via extraluminal placement, avoiding direct contact between the stent and airway mucosa. This technique substantially reduces mucosal irritation and inflammatory complications with stable fixation, making it particularly suitable for pediatric patients and cases of extrinsic compressive tracheobronchomalacia (12).

The biodegradable PLLA microplate used in this case offers several advantages for pediatric airway application. First, PLLA is a Food and Drug Administration-approved biodegradable polymer with excellent histocompatibility and has been widely used in cardiovascular, orthopedic, and reconstructive surgery. Second, it provides high flexural strength (190 MPa) for reliable airway support. Third, it exhibits gradual, controllable *in vivo* degradation: mechanical support diminishes 7–10 months after implantation, complete biodegradation is achieved within 3–5 years, and the final degradation products are carbon dioxide and water, which do not interfere with pediatric airway growth and development.

This patient had congenital heart disease. Echocardiography revealed left atrial enlargement. Combined with three-dimensional reconstruction of chest CT and bronchoscopy findings, the diagnosis of acquired tracheomalacia with severe tracheal collapse secondary to tracheal compression caused by cardiomegaly was confirmed. Intracardiac malformation repair under cardiopulmonary bypass combined with simultaneous bronchial splint implantation resulted in immediate symptomatic relief and substantial resolution of tracheal stenosis. No major complications occurred during the 2-year follow-up period, and normal growth and development indicated favorable therapeutic outcomes.

In conclusion, pediatric tracheomalacia induced by cardiomegaly in patients with heart disease is a rare clinical entity that is frequently misdiagnosed as simple heart failure. The combination of bronchoscopy and three-dimensional chest CT reconstruction is key to a definitive diagnosis. Bronchial splint implantation is a safe and effective treatment for such patients and can substantially improve ventilation with satisfactory long-term tolerance, thereby offering a vital therapeutic alternative. Nevertheless, owing to the limited number of relevant cases, further clinical studies are required to validate long-term efficacy.

## Data Availability

The original contributions presented in the study are included in the article/Supplementary Material; further inquiries can be directed to the corresponding authors.
